# *Prototheca* spp. induce an inflammatory response via mtROS-mediated activation of NF-κB and NLRP3 inflammasome pathways in bovine mammary epithelial cell cultures

**DOI:** 10.1186/s13567-021-01014-9

**Published:** 2021-12-11

**Authors:** Wenpeng Zhao, Fumeng He, Herman W. Barkema, Siyu Xu, Jian Gao, Gang Liu, Zhaoju Deng, Muhammad Shahid, Yuxiang Shi, John P. Kastelic, Bo Han

**Affiliations:** 1 Department of Clinical Veterinary Medicine, College of Veterinary Medicine, China Agricultural University, Beijing, 100193 China; 2 Department of Production Animal Health, Faculty of Veterinary Medicine, University of Calgary, Calgary, AB T2N 4N1 Canada; 3 College of Life Sciences and Food Engineering, Hebei University of Engineering, Handan, 056038 Hebei China

**Keywords:** *Prototheca* spp., bovine mammary epithelial cells, mtROS, NF-κB/NLRP3 inflammasome pathway, inflammation

## Abstract

Emergence of bovine mastitis caused by *Prototheca* algae is the impetus to better understand these infections. Both *P. bovis* and *P. ciferrii* belong to *Prototheca* algae, but they differ in their pathogenicity to induce inflammatory responses. The objective was to characterize and compare pathogenesis of inflammatory responses in bMECs induced by *P. bovis* versus *P. ciferrii.* Mitochondrial ultrastructure, activity and mtROS in bMECs were assessed with transmission electron microscopy and laser scanning confocal microscopy. Cytokines, including TNF-α, IL-1β and IL-18, were measured by ELISA and real-time PCR, whereas expressions of various proteins in the NF-κB and NLRP3 inflammasome pathways were detected with immunofluorescence or Western blot. Infection with *P. bovis* or *P. ciferrii* damaged mitochondria, including dissolution and vacuolation of cristae, and decreased mitochondrial activity, with *P. bovis* being more pathogenic and causing greater destruction. There were increases in NADPH production and mtROS accumulation in infected bMECs, with *P. bovis* causing greater increases and also inducing higher cytokine concentrations. Expressions of NF-κB-p65, p-NF-κB-p65, IκBα and p-IκBα proteins in the NF-κB pathway, as well as NLRP3, Pro Caspase1, Caspase1 p20, ASC, Pro IL-1β, and IL-1β proteins in the NLRP3 inflammasome pathway, were significantly higher in *P. bovis*-infected bMECs. However, mito-TEMPO significantly inhibited production of cytokines and decreased expression of proteins in NF-κB and NLRP3 inflammasome pathways in bMECs infected with either *P. bovis* or *P. ciferrii*. In conclusion, *P. bovis* or *P. ciferrii* infections induced inflammatory responses in bMECs, with increased mtROS in damaged mitochondria and activated NF-κB and NLRP3 inflammasome pathways, with *P. bovis* causing a more severe reaction.

**Supplementary Information:**

The online version contains supplementary material available at 10.1186/s13567-021-01014-9.

## Introduction

Mastitis is common in dairy cattle worldwide, causing serious reductions in milk yield and quality and large financial losses [1, 2]. Infections with pathogens are an important cause of mastitis. *Prototheca* spp. are unicellular achlorophyllous algae, 3–30 µm in diameter, that lack a specific glucosamine cell wall or chloroplasts; specific species include *P. bovis*, *P. ciferrii*, *P. cerasi*, *P. pringsheimii*, *P. blaschkeae*, *P. wickerhamii*, *P. xanthoriae*, *P. cookie*, *P. xanthoriae*, *P. cutis*, *P. miyajii*, *P. tumulicola*, *P. moriformis*, *P. stagnora*, and *P. ulmea* [3–5]. Bovine mastitis caused by *Prototheca* spp. is characterized by an abrupt decrease in both milk production and quality, an increased somatic cell count, and frequently culling, with substantial economic losses [6]. Among *Prototheca* spp., *P. bovis* was the causative pathogen of bovine mastitis, whereas *P. ciferrii* occasionally causes granulomatous lesions in experimentally infected bovine udders and protothecosis in humans [7, 8]. *Prototheca* spp. mastitis has been reported in many countries, including Canada, Poland, Italy, Brazil, and Japan [9–11].

An inflammatory response, a typical feature of bovine mastitis, is characterized by release of inflammatory cytokines such as IL-1β, TNF-α and IL-18. Numerous signal molecules or pathways are involved in regulation of an inflammatory response, including reactive oxygen species, inflammasome and NF-κB pathway [12, 13]. The inflammasome is an upstream regulatory mechanism that triggers an inflammatory response when stimulated by pathogens. The best characterized inflammasome is the NLRP3 inflammasone, comprised of NLRP3, apoptosis-associated speck-like protein containing adaptor (ASC), and Caspase1 [12–14]. Furthermore, NLRP3 is a cytosolic pattern recognition receptor (PRR) activated by pathogen-associated molecular patterns (PAMPs) and damage-associated molecular patterns (DAMPs) [15]. Once activated, the inflammasome recruits NLRP3, ASC, and Caspase1, and cleaves Pro Caspase1 to an active form (cleavage Caspase1) that triggers proteolytic cleavage of Pro IL-1β and Pro IL-18 to mature and secreted forms [12, 15]. Although NLRP3 signaling usually confers protection, excessive activation can damage cells and cause inflammatory diseases [16–18]. The NLRP3 inflammasome can be generated and activated by *Escherichia coli* and *Staphylococcus aureus*, causing an aggravated inflammatory response and damage in bMECs [19, 20]. Activation of the NLRP3 inflammasome is regulated by various genes. In that regard, NF-κB participates, and has an important regulatory role, in activation of the NLRP3 inflammasome, which triggers an inflammatory response [21]. Furthermore, an activated NF-κB pathway could function as an upstream activator of NLRP3 and contribute to regulating inflammatory cytokines [22, 23].

Mitochondrial reactive oxygen species (mtROS) also activate the NLRP3 inflammasome, promoting inflammation and enhancing immune responses [24, 25]. Accumulation of damaged mitochondria may be essential for NLRP3 activation. In addition to increased mtROS, exposure of mitochondria-derived DAMPs (mtDAMPs) [e.g., mitochondrial DNA (mtDNA)] and cardiolipin to the cytosol, can also promote NLRP3 activation [26, 27]. Activation of the NLRP3 inflammasome has a crucial role in inflammatory responses in many diseases. Clinical bovine mastitis is usually characterized by pain, edema, cytokine production, and cellular infiltration. In *Prototheca* spp. mastitis, there are interstitial infiltrates of macrophages, plasma cells and lymphocytes into the mammary gland, and an antiserum against bovine keratin had weak positive expression in damaged mammary tissue [28]. We reported that infections with *P. bovis* or *P. ciferrii* increased expression of cytokine mRNA in bMECs [29]; however, inflammatory responses in bovine mammary epithelial cells (bMECs) infected with *P. bovis* or *P. ciferrii* are not well characterized. Therefore, mitochondrial damage, inflammatory cytokines including TNF-α, IL-1β and IL-18, and protein expression in the NF-κB/NLRP3 pathway that regulate inflammation were measured to characterize and compare the pathogenesis of inflammatory responses in bMECs induced by infection with *P. bovis* versus *P. ciferrii.*

## Materials and methods

### Reagents and antibody

Cell Counting Kit-8 (CCK-8), NADP + /NADPH assay kit, Bicinchoninic acid (BCA) protein assay kit, radioimmunoprecipitation assay (RIPA) lysis buffer, Mito-Tracker Green staining solution and Hoechst 33342 live cell staining solution were purchased from Beyotime (Shanghai, China). ELISA assay kit was purchased from mlbio (Shanghai, China). 4’, 6-Diamidine-2’-phenylindole dihydrochloride (DAPI), coverslips and Triton X-100, penicillin, streptomycin and bovine serum albumin (BSA) was purchased from Solarbio (Beijing, China). Enhanced chemiluminescence (ECL) kits were obtained from Thermo Fisher Scientific Pierce (Rockford, IL, USA). Fetal Bovine Serum (FBS) and Dulbecco’s Modified Eagle’s medium (DMEM) were purchased from Hyclone (Logan, UT, USA). Rotenone was purchased from MCE (Shanghai, China). Mito-SOX red mitochondrial superoxide indicator was purchased from Yeasen (Shanghai, China). Trizol Reagent, cDNA synthesis superMix and Two-step RT-PCR superMix were purchased from TransGen Biotech (Beijing, China). Primary antibodies, including NLRP3, ASC, Caspase-1, IL-1β and α-Tubulin, were purchased from Proteintech (Wuhan, China), and NF-κB p65, Phospho-NF-κB p65, IκBα and Phospho-IκBα were purchased from Cell Signaling Technology (Danvers, MA, USA). Peroxidase-conjugated goat anti-mouse IgG and goat anti-rabbit IgG were purchased from Proteintech (Wuhan, China).

### *P. bovis* and *P. ciferrii* isolates

*Prototheca bovis* was isolated in 2016 from 105 clinical mastitis milk samples collected on 6 large (> 500 cows) Chinese dairy farms, whereas the 58 *P. ciferrii* isolates were recovered in the same year from environmental samples from 3 large dairy farms, located in suburbs of Beijing, Tianjin and Shandong [3]. The isolates were stored at 4 °C at the College of Veterinary Medicine, China Agricultural University, Beijing, China [3]. These *Prototheca* spp. were characterized as *P. bovis* and *P. ciferrii* by several methods. Firstly, based on cellular fatty acid pattern, *P. bovis* had more eicosadienoic acid (C20:2) compared to *P. ciferrii* [7]. Secondly, we determined 18S rDNA sequences using genotype-specific PCR. For this, a PCR mix (20 µL) containing *Prototheca* (450 bp) fragment internal amplification control *Proto*18-4f (GACATGGCGAGGATTGACAGA) and *Proto*18-4r (AGCACACCCAATCGGTAGGA) primers (2.5 µL each primer), DNA template (1 µL), ddH_2_O (4 µL), and 2 × EasyTaq PCR supermix (10 µL) was amplified under specific conditions (2 min at 95 °C, followed by 34 cycles of 30 s at 95 °C, 30 s at 50 °C, and 30 s at 72 °C, with a final extension of 5 min at 72 °C). Amplified fragments were sent for sequencing (Sangon Biotech, Shanghai, China). Then, *P. bovis* and *P. ciferrii* were characterized by genotype-specific primers [3, 7]. Additionally, the *P. bovis* and *P. ciferrii* genotypes were further confirmed by restriction fragment length polymorphism analysis targeting the *cytb* gene fragment [7, 30]. Taken together, we confirmed *P. bovis* and *P. ciferrii* genotypes in the isolates recovered from clinical mastitis milk and environmental samples. These strains in within a species (*P. bovis* and *P. ciferrii*), strains had the same genotype, colony morphology and similar biochemical characteristics. We randomly selected 3 strains of each species for the following experiments, which were performed independently in triplicate. The 3 strains of *P. bovis* and 3 strains of *P. ciferrii* were isolated from clinical mastitis milk and environmental samples of 3 large farms located near suburbs of Beijing, Tianjin and Shandong, respectively [3]. Furthermore, within each species, genotype and colony morphology were the same and the biochemical characteristics were similar. Additionally, *P. ciferrii* grew more slowly than *P. bovis* on SDA and their colonies had differences in morphological characteristics; *P. ciferrii* produced small colonies with smooth surface and folded edges compared to *P. bovis*, whereas the latter had more eicosadienoic acid (C20:2) compared to the former [7]. *P. bovis* and *P. ciferrii* isolates were multiplied by streaking on sabouraud dextrose agar (SDA) and incubated at 37 °C for 48 h. Then, a single colony was placed in sabouraud dextrose broth (SDB) and incubated for 72 h. Thereafter, organisms were diluted in DMEM to achieve required concentrations.

### Cell culture and treatment

The MAC-T line of bMECs (Shanghai Jingma Biological Technology Co., Ltd. China) was used for cell culture. bMECs were placed in DMEM medium supplemented with 10% fetal bovine serum, penicillin (100 U/mL) and streptomycin (100 U/mL) and grown in cell culture plates. Cells were incubated in 5% CO_2_ at 37 °C, and cells from passages 2–8 were used for experiments. Before infection, cells were put in 6-well plates (1 × 10^6^ cells per well) and cultured overnight. Next, cells were infected with *P*. *bovis* or *P*. *ciferrii* at a 5:1 multiplicity of infection (MOI; ratio of *P*. *bovis* or *P*. *ciferrii* to bMECs) and incubated in 5% CO_2_ at 37 °C for 12 h. Then, culture supernatants were collected and frozen (−80 °C) to subsequently determine cytokine concentrations, whereas cells were collected to extract and characterize proteins. Each experiment was conducted in triplicate.

### Transmission electron microscopy

The bMECs were fixed as described [29] and transmission electron microscopy (TEM) used to assess ultrastructure. Briefly, cells were washed 3 times with phosphate buffered solution (PBS) and then fixed with 2.5% glutaraldehyde solution (pH 7.4) for 2–4 h at room temperature. After fixation, samples were routinely processed and examined with a transmission electron microscope (H7650, Hitachi, Tokyo, Japan) at an accelerating voltage of 80 kV.

### Cell viability assay

Cell viability was measured with a Cell Counting Kit-8 (CCK-8). The bMECs were seeded into 96-well plates at a density of 5 × 10^3^ cells/well, allowed to adhere overnight, and then treated for 12 h with various concentrations of rotenone (mitochondrial electron transport chain complex I inhibitor) used to enhance mitochondrial reactive oxygen species production (i.e., a positive control). Then, bMECs were washed 3 times with PBS and 10 µL CCK-8 solution added to each well. After incubation for 1.5 h at 37 °C with 5% CO_2_, OD values were read at 570 nm.

### Mito-tracker green staining

The bMECs were cultured in 6-well plates overnight and then infected with *P. bovis* or *P. ciferrii* at a 5:1 MOI. After 12 h, bMECs were washed 3 times with PBS and 2 mL of warm (37 °C) Mito-Tracker Green staining solution was added. After incubation for 30 min at 37 °C, Mito-Tracker Green staining solution was removed and 2 mL fresh cell culture solution (37 °C) was added. Then, 10 μL Hoechst 33342 live cell staining solution was added to each well. After incubating for 10 min at 37 °C, the dye-containing culture medium was aspirated, cells were washed 3 times with culture medium and observed with laser scanning confocal microscopy (Olympus-FV3000, Olympus, Tokyo, Japan).

### Mitochondrial ROS measurement

To detect intracellular mtROS production, bMECs were seeded into 6-well plates with cell climbing films and infected with *P*. *bovis* or *P*. *ciferrii* at a 5:1 MOI. After 12 h, Mito-SOX red mitochondrial superoxide indicator was used to label mitochondrial reactive oxygen species. To induce accumulation of mtROS (positive control), bMECs were treated with 2.5 μM rotenone for 12 h. Next, cells were incubated with Mito-SOX (5 μM) in the dark for 10 min at 37 °C and then washed 3 times with PBS. Nuclei were stained with 300 nM 4. 6-diamimo-2-phenyl indole (DAPI) for 5 min at 37 °C and washed with PBS. Slides were covered with glass cover slips and intracellular mtROS assessed with laser scanning confocal microscopy (Olympus-FV3000).

### NADPH analysis

The bMECs were cultured into 6-well plates overnight and then infected with *P. bovis* or *P. ciferrii* at a 5:1 MOI for 12 h. The NADPH content in cells was determined with a commercial NADP + /NADPH Assay Kit, according to the manufacturer’s protocol. Briefly, 200 μL NADP + /NADPH extract was added into each hole of the 6-well plate, gently blown to promote cell lysis, and supernatant collected for subsequent experiments. Then, 50 μL supernatant and 200 μL of G6PDH working solution were added into each 96-well plate. After incubation for 10 min at 37 °C, 10 μL chromogenic solution was added into each well and after incubation for 20 min at 37 °C, absorbance was measured at 450 nm.

### ELISA

The bMECs were infected with the 3 *P. bovis* or 3 *P. ciferrii* isolates at a 5:1 MOI for 12 h, and 10 µM of 2-(2,2,6,6-Tetramethylpiperidin-1-oxyl-4-ylamino)-2-oxoethyl) triphenyl-phosphonium chloride, monohydrate (mito-TEMPO), a mitochondria-targeted superoxide dismutase mimetic with superoxide and alkyl radical scavenging properties, was used to scavenge superoxide. Cytokines in supernatants of bMECs culture medium were quantified by ELISA kits, according to the manufacturer’s instructions. Cell culture supernatants were collected and concentrations of TNF-α, IL-1β and IL-18 in supernatants were measured, based on OD values at 450 nm.

### RNA extraction and real time PCR

The bMECs were treated as described above for ELISA, washed 3 times with PBS, and cells collected for total RNA extraction. Trizol Reagent was pre-chilled on ice and 1 mL added to cell samples for 5 min to lyse cells. Mixed liquid was centrifuged at 12 000 × *g* for 15 min at 4 °C and supernatant collected. Total mRNA of bMECs was extracted with mRNA extraction kit according to manufacturer’s instructions. Relative expression levels of TNF-α, IL-1β and IL-18 mRNA were determined using the StepOnePlus Real-Time PCR systems. Data were analyzed according to the 2^−∆∆Ct^ method and results were expressed as relative mRNA levels [7]. Primer sequences for GAPDH (housekeeping gene), TNF-α, IL-1β, and IL-18 are presented in Table 1.

**Table 1:** **List of primers for real-time PCR.**

Gene	Primer	Sequence (5’-3’)	Size (bp)
GAPDH	Forward Reverse	TCACCAACTGGGACGACA GCATACAGGGACAGCACA	206
TNF-α	Forward Reverse	ATGTGTGTGGAGAGCGTCAA GGGCCATACAGCTCCACAAA	145
IL-1β	Forward Reverse	ATGACTTCCAAGCTGGCTGTTG TTGATAAATTTGGGGTGGAAAG	114
IL-18	Forward Reverse	TTGCATCAGCTTTGTGGAAA TGGGGTGCATTATCTGAACA	213

### Immunofluorescence

The bMECs were treated as described above for ELISA, washed 3 times with PBS and then fixed in 4% paraformaldehyde for 30 min and subsequently permeabilized in 0.25% Triton X-100. Cells were incubated with 3% bovine serum for 30 min at room temperature and then incubated overnight at 4 °C with the following primary antibodies: NLRP3, NF-κB p65, Phospho-NF-κB p65, ASC, and IL-1β. Next, samples were washed with PBS and incubated with Alexa Fluor 488-labeled goat anti-rabbit IgG (H + L) for 1 h at room temperature. Then, samples were washed with PBS and stained with DAPI for 20 min. After washing with PBS, slides were covered with glass cover slips and observed under a laser scanning confocal microscope (Olympus-FV3000).

### Western blot

The bMECs were treated as described above for ELISA and then lysed on ice and the cell lysate suspension collected and centrifuged (12 000 × *g*, 4 °C) for 15 min. Total protein concentration in the supernatant was determined with a BCA protein assay kit. Protein samples were denatured in boiling water for 10 min and then separated by SDS-PAGE and transferred onto polyvinylidene difluoride (PVDF) membranes. These membranes were blocked with 5% nonfat dry milk for 2 h at room temperature, then incubated overnight at 4 °C with the following primary antibodies: α-Tubulin, NLRP3, NF-κB p65, Phospho-NF-κB p65, IκBα, Phospho-IκBα, ASC, Caspase-1, and IL-1β. For α-Tubulin, membranes were incubated with mouse anti-α-Tubulin antibody, whereas for all other proteins, they were incubated with secondary antibody against rabbit IgG for 1 h at room temperature. After washing with Tris-buffered saline, the membrane was developed using ECL reagents and visualized with a chemiluminescence system. Results were normalized to α-Tubulin, and band density was analyzed with Image J (National Institutes of Health, Bethesda, MD, USA).

### Statistical analyses

After visually confirming that the data were normally distributed, independent Student’s *t*-tests or one-way ANOVA were used to analyze effects of *Prototheca* spp. on cell viability, NADPH content, inflammatory cytokines including TNF-α, IL-1β and IL-18, and protein expression in NF-κB/NLRP3 pathway, with a Bonferroni method used to correct multiple comparisons, *P* < 0.05 was divided by the number of tests to be considered statistically significant. Data are reported as means ± standard deviation (SD) of 3 independent experiments (3 technical replicates were carried out in each experiment).

## Results

### *Prototheca* spp. infection caused mitochondrial damage in bMECs

**Figure 1:**
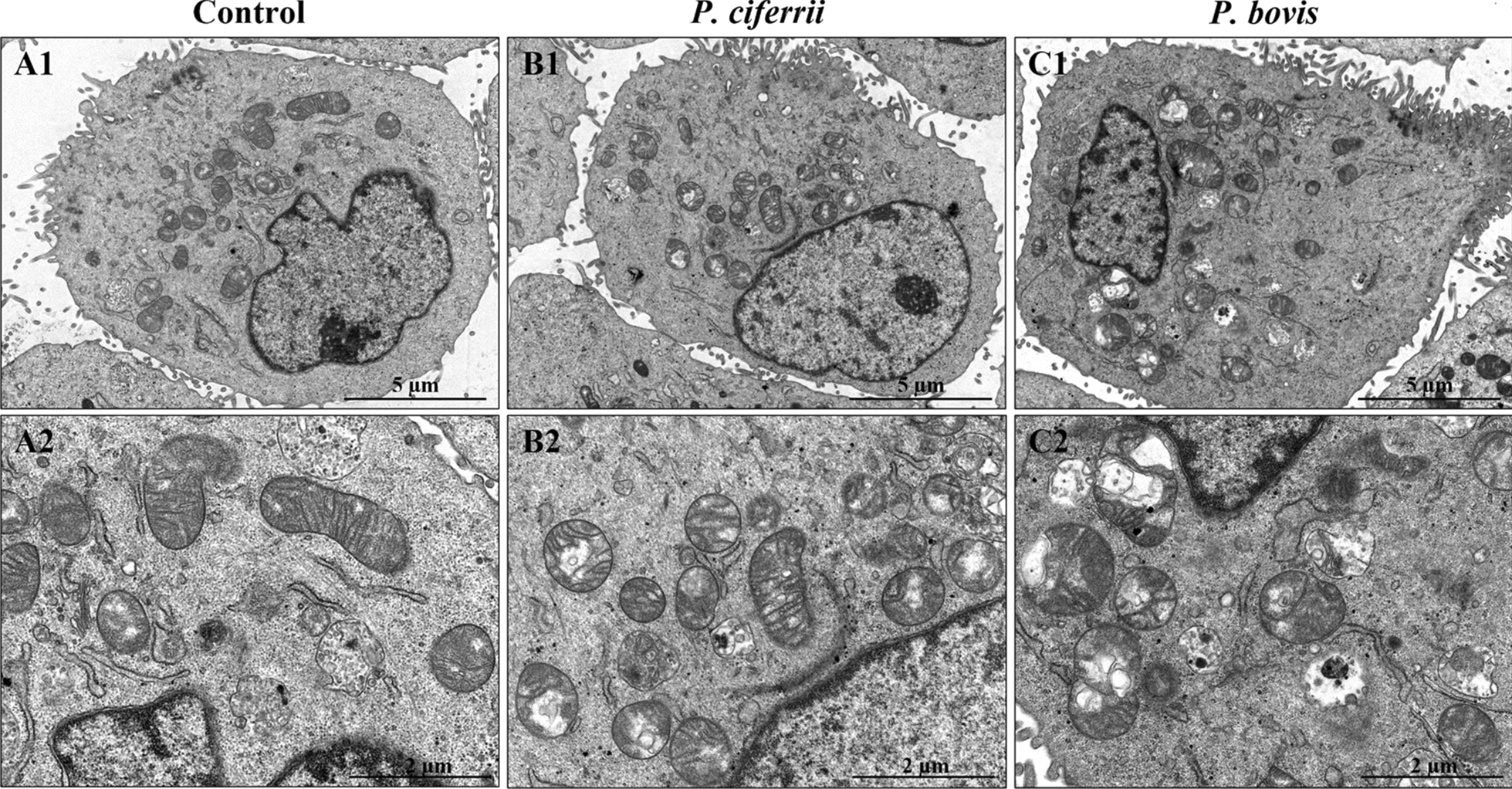
**Mitochondrial ultrastructure in bMECs.** **A1** and **A2**: Control group, with normal mitochondria and mitochondrial cristae in the bMECs. **B1** and **B2**: *P. ciferrii* infection group; note the slight vacuolization in mitochondria in *P. ciferrii-*infected bMECs. **C1** and **C2**: *P. bovis* infection group; note the mitochondrial cristae dissolution and large areas of vacuolation in bMECs.

**Figure 2:**
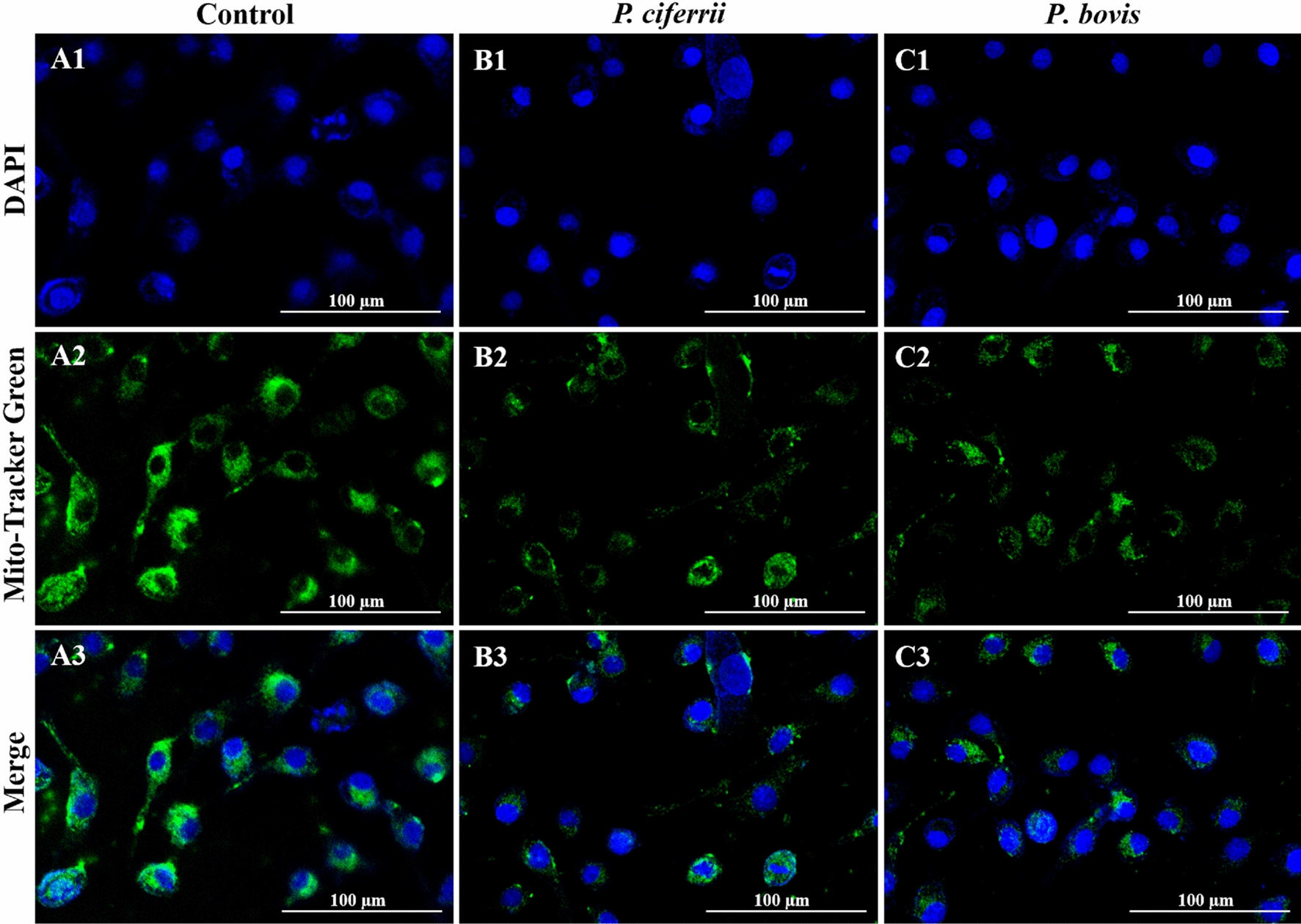
**Mitochondrial activity in bMECs.** Mito-Tracker Green is a mitochondrial green fluorescent probe, with intensity of green fluorescence reflecting mitochondrial activity. **A1**, **A2**, and **A3**: In the Control group, mitochondria in normal bMECs had strong green fluorescence, indicating good mitochondrial activity. **B1**, **B2**, and **B3**: In the *P. ciferrii* infection group, there was weak green fluorescence, indicating decreased mitochondrial activity in bMECs. **C1**, **C2**, and **C3**: In the *P. bovis* infection group, there was weak green fluorescence in bMECs, indicating mitochondrial activities were decreased.

In uninfected bMECs (controls), mitochondria had reticulated morphologies and mitochondrial cristae were clearly visible with TEM (Figure 1, panels A1 and A2), whereas in bMECs infected with *P. ciferrii,* mitochondria had relatively minor damage, including slight vacuolization (Figure 1, panels B1 and B2). In contrast, in bMECs infected with *P. bovis*, mitochondria had dissolution of their cristae and large areas of vacuolation (Figure 1, panels C1 and C2). Intensity of green fluorescence was profoundly decreased in bMECs infected with *P. bovis,* with less suppression in the *P. ciferrii* infection group, although both were lower than the control (Figure 2), indicating decreased mitochondrial activity in infected bMECs. Both *P. bovis* and *P. ciferrii* induced mitochondrial damage, with more severe damage caused by *P. bovis*.

### *Prototheca* spp. infection enhanced mtROS accumulation in bMECs

**Figure 3:**
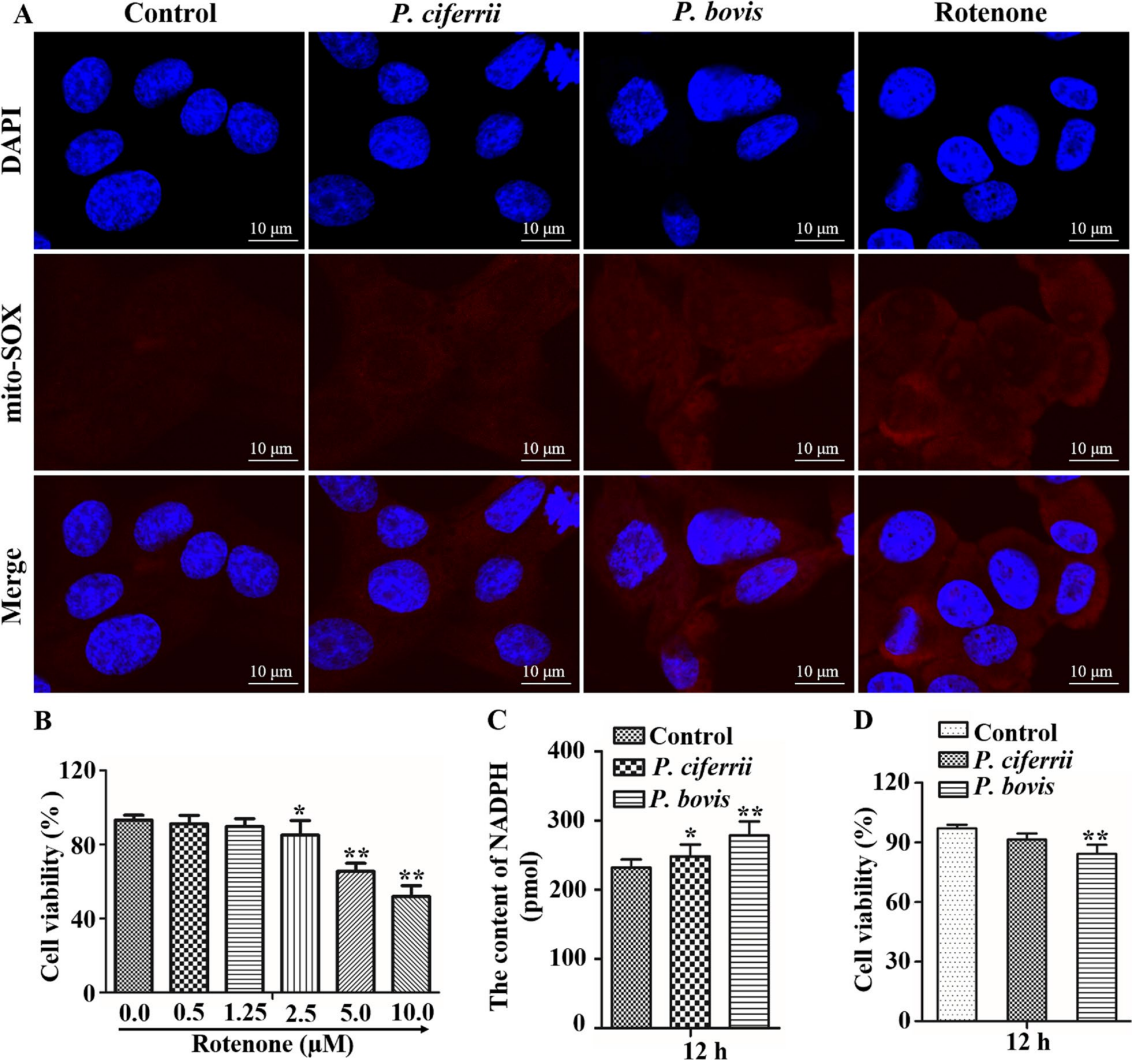
**Mitochondrial accumulation of reactive oxygen species in bMECs.** **A** Mit-SOX red mitochondrial superoxide indicator (5.0 µM) was used to label reactive oxygen species in mitochondria (mtROS) of bMECs infected with *P. bovis* or *P. ciferrii*; both induced mtROS accumulation, with *P. bovis* being more severe*.* Rotenone (2.5 µM), a mitochondrial electron transport chain complex I inhibitor, was used to induce production of mtROS. **B** Effects of various concentrations of rotenone on bMECs viability (note the gradual decrease with increasing rotenone concentrations). As a positive control, bMECs were treated with 2.5 µM rotenone. **C** NADPH in bMECs infected with either *P. bovis* or *P. ciferrii*. **D** Cell viability in infected bMECs. Data represent means ± SD of 3 independent experiments. ^*^P < 0.05 or ^**^P < 0.01, difference compared to the control.

In bMECs infected with *P. ciferrii,* the mtROS assay had weak red fluorescence (Figure 3A). As a positive control, bMECs were treated with various concentrations of rotenone, with 2.5 µM rotenone used to treat bMECs (Figure 3B). However, strong red fluorescence was observed in bMECs infected with *P. bovis* or treated with rotenone (Figure 3A); therefore, *P. bovis* induced greater mtROS accumulation. Furthermore, NADPH content was higher in bMECs infected with *P. bovis* or *P. ciferrii* compared to the control (*P* < 0.05), with the highest NADPH in the *P. bovis* infection group (Figure 3C). *P. bovis* and *P. ciferrii* decreased bMECs viability at 12 h post infection, although *P. bovis* caused a more profound decrease than *P. ciferrii* in the viability of bMECs (Figure 3D).

### *Prototheca* spp. infection increased production of TNF-α, IL-1β and IL-18 in bMECs

**Figure 4:**
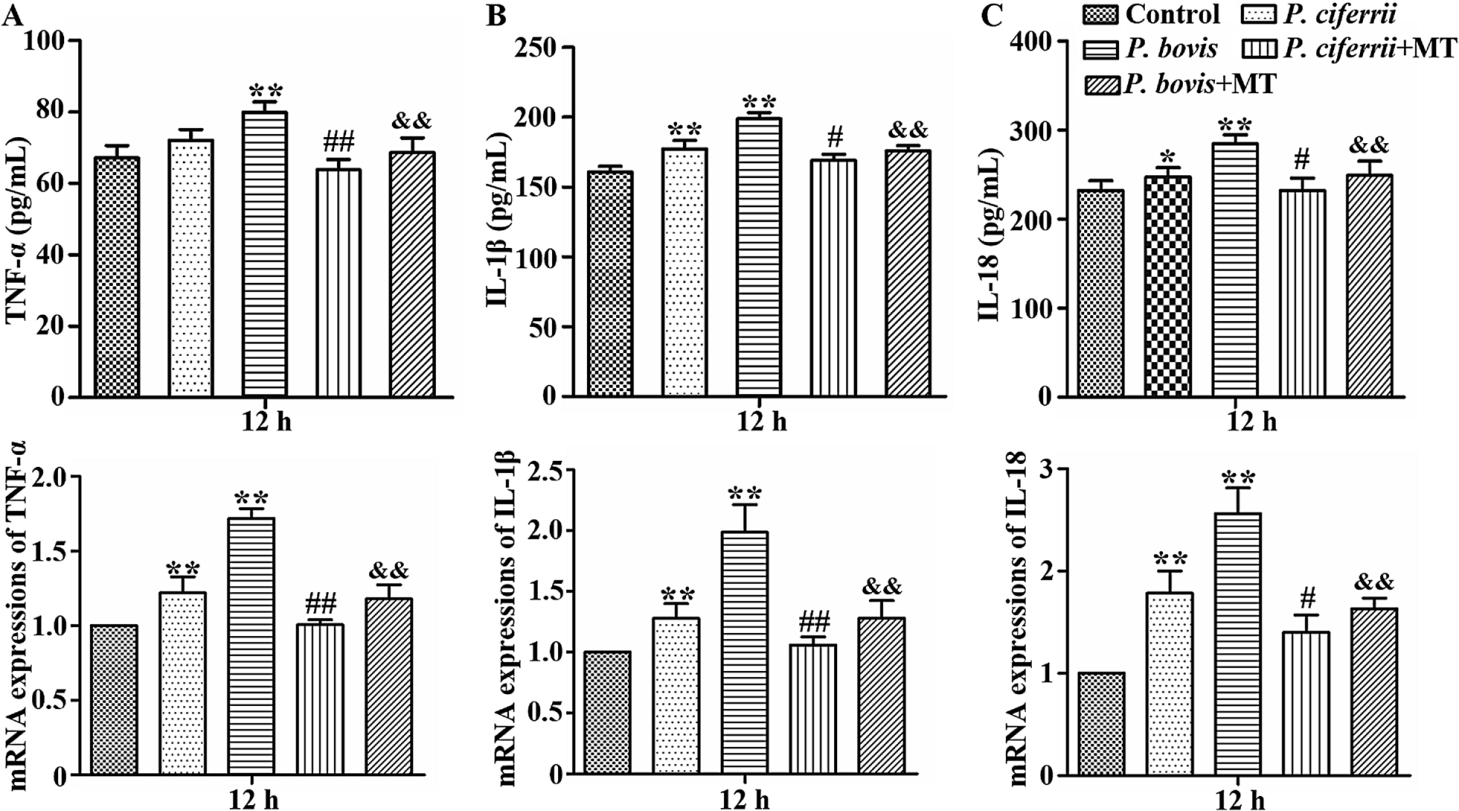
**Production of TNF-α, IL-1β, and IL-18 in bMECs.** **A**, **B** and **C** Changes of TNF-α, IL-1β and IL-18 in bMECs measured by ELISA and real time PCR, respectively. Treatment with MT (10 µM) for 12 h inhibited cytokine production in bMECs infected with *P. bovis* or *P. ciferrii*. The mRNA expression was analyzed according to the 2^−∆∆Ct^ method and results were expressed as relative mRNA levels. Data represent means ± SD of 3 independent experiments. ^*^P < 0.05 or ^**^P < 0.01, difference compared to the control; ^#^*P* < 0.05 or ^##^*P* < 0.01, difference compared to the *P. ciferrii* infection group; ^&^*P* < 0.05 or ^&&^*P* < 0.01, difference compared to the *P. bovis* infection group.

Infection with *P. ciferrii* increased production of IL-1β, and IL-18 proteins and mRNAs in bMECs (*P* < 0.05), with more profound increases in bMECs infected with *P. bovis* (*P* < 0.05) (Figures 4B and C). *P. bovis* infection increased production of TNF-α proteins and mRNAs in bMECs (*P* < 0.05) (Figure 4A). In contrast, treatment with mito-TEMPO inhibited production of TNF-α, IL-1β and IL-18 proteins and mRNAs in bMECs infected with *P. bovis* or *P. ciferrii* (*P* < 0.05) (Figures 4A, B and C). Expression of TNF-α, IL-1β and IL-18 was not significantly different among isolates within *P. bovis* nor among *P. ciferrii* species (Additional file 1).

### *Prototheca* spp. infection promoted protein expression of NF-κB pathway in bMECs

**Figure 5:**
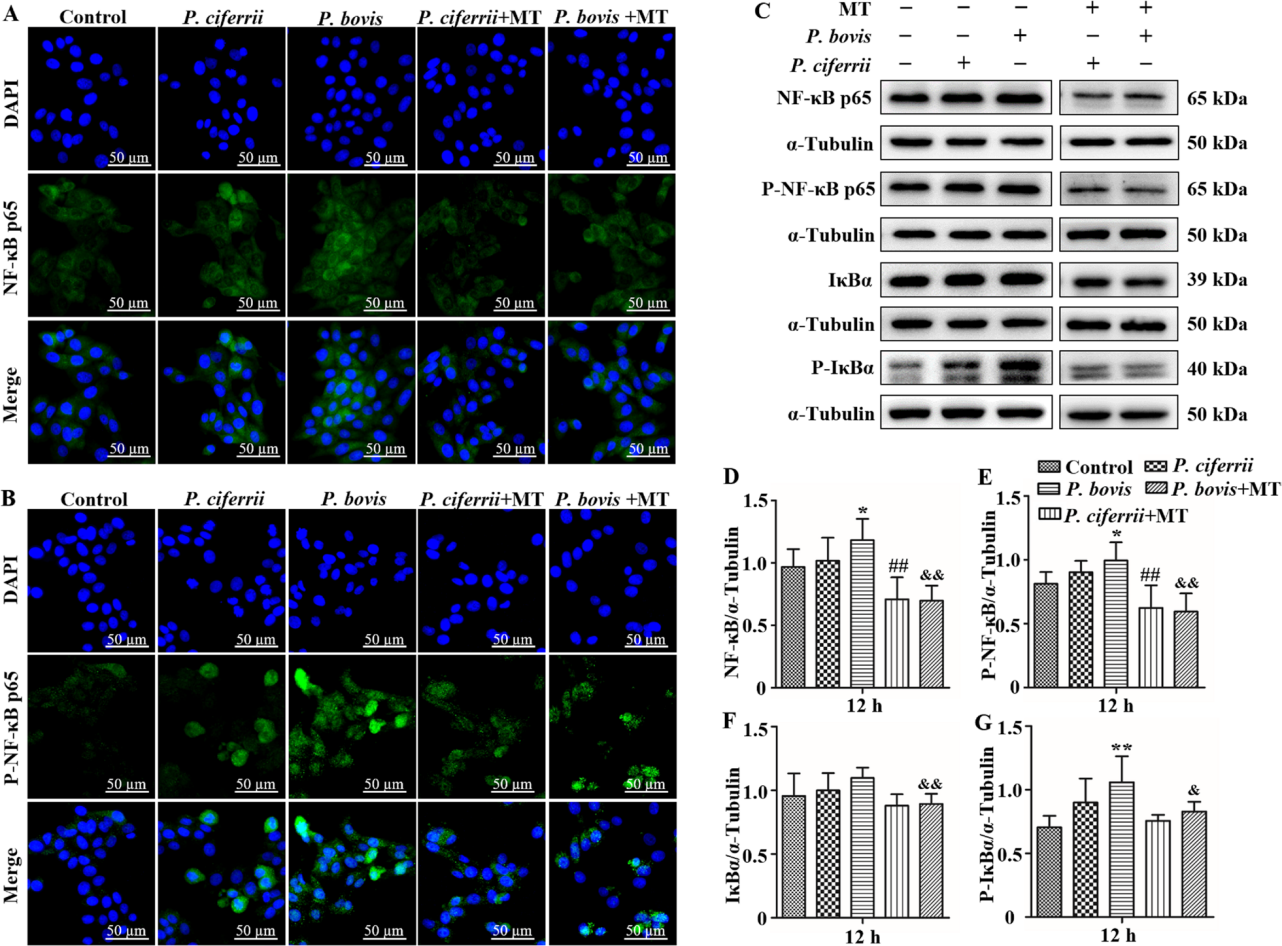
**Expression of NF-κB pathway proteins in bMECs.** **A** and **B** Green fluorescence is expression of NF-κB p65 and p-NF-κB p65 proteins; treatment with MT (10 µM) for 12 h inhibited expression of these proteins in bMECs infected with *P. bovis* or *P. ciferrii*. **C**–**G** Treatment with MT (10 µM) for 12 h reduced expression levels of NF-κB p65, p-NF-κB p65, IκBα, and p-IκBα proteins in bMECs infected with *P. bovis* or *P. ciferrii*. “-” and “ + ” after MT indicated that MT was not or was added, respectively; “-” and “ + ” after *P. bovis* indicated that *P. bovis* was not or were added, respectively; “-” and “ + ” after *P. ciferrii* indicated that *P. ciferrii* was not or were added, respectively. Data represent means ± SD of 3 independent experiments. ^*^P < 0.05 or ^**^P < 0.01, difference compared to the control; ^#^*P* < 0.05 or ^##^*P* < 0.01, difference compared to the *P. ciferrii* infection group; ^&^*P* < 0.05 or ^&&^*P* < 0.01, difference compared to the *P. bovis* infection group.

Infection with *P. bovis* increased the green fluorescence intensity of NF-κB p65 and p-NF-κB p65 in bMECs (Figures 5A and B). Furthermore, in Western blots, protein expression levels of NF-κB p65 and p-NF-κB p65 were also upregulated in *P. bovis*-infected bMECs (*P* < 0.05) compared to the control (Figures 5C, D and E). Although *P. ciferrii* infection in bMECs also increased expression levels of NF-κB p65 and p-NF-κB p65 proteins, changes were less profound than in the *P. bovis* infection group, with the immunofluorescence consistent with the Western blot (Figures 5A, B, C, D and E). In addition, expression level of p-IκBα proteins was upregulated in *P. bovis-*infected cells (*P* < 0.05); there were fewer profound increases induced by *P. ciferrii*, but p-IκBα (*P* > 0.05) was higher than the control (Figures 5C and G). Mito-TEMPO inhibited expression levels of NF-κB p65 and p-NF-κB p65 proteins in bMECS infected with *P. bovis* or *P. ciferrii* (*P* < 0.05) (Figures 5C, D and E). A similar trend in expression levels of IκBα and p-IκBα protein in bMECs infected with *P. bovis* or *P. ciferrii* was also observed after treatment with mito-TEMPO, but IκBα and p-IκBα were decreased compared to the *P. bovis* group (*P* < 0.05) (Figures 5C, F and G). Protein expression in the NF-κB pathway was not significantly different among isolates within *P. bovis* or *P. ciferrii* species (Additional file 2).

### *Prototheca *spp*.* infection contributed to NLRP3 inflammasome activation in bMECs

**Figure 6:**
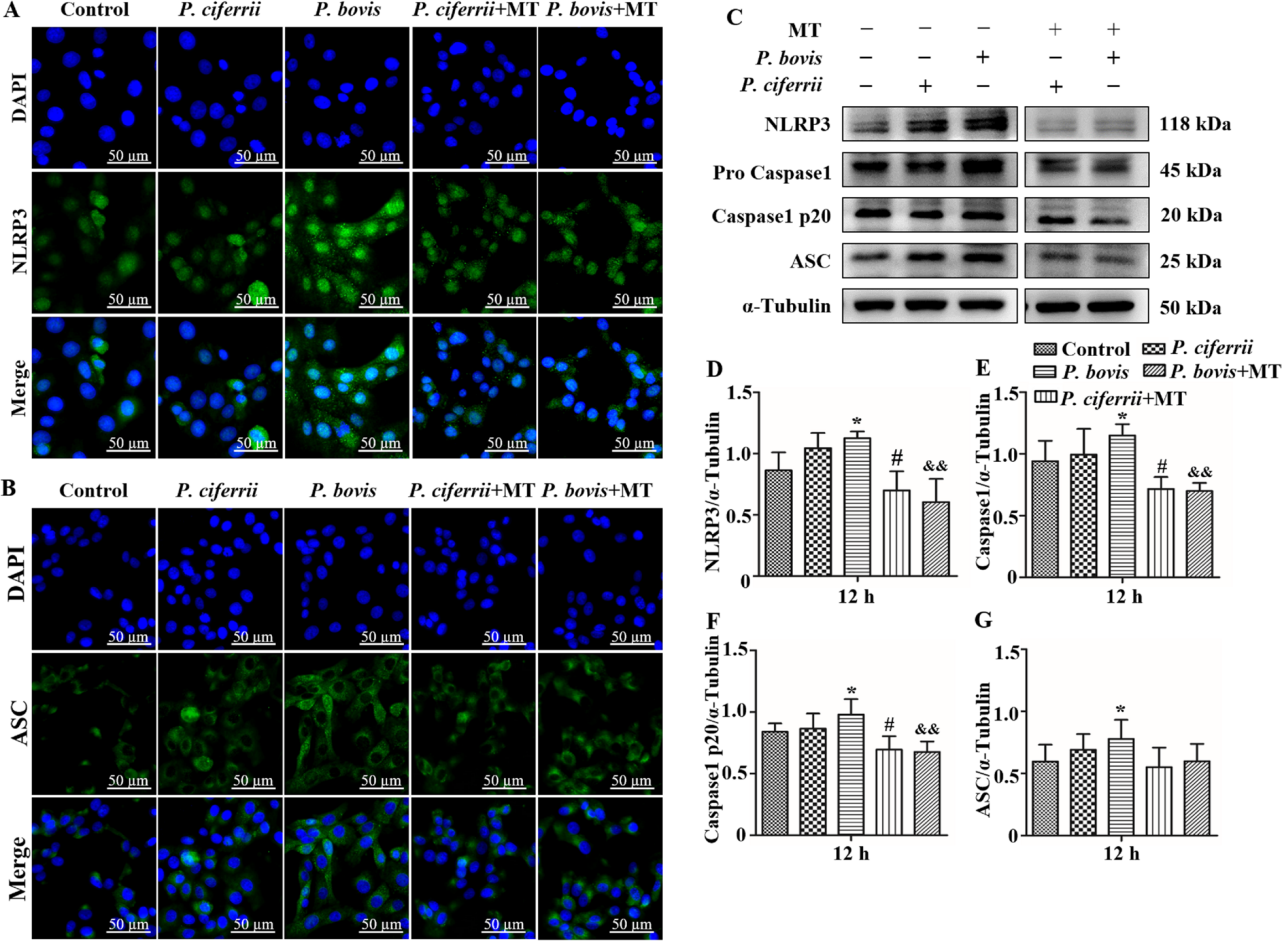
**Expression of NLRP3 inflammasome pathway proteins in bMECs.** **A** and **B** in *P. bovis-* or *P. ciferrii*-infected bMECs. Treatment with MT (10 µM) for 12 h inhibited protein expression of NLRP3 and ASC in *P. bovis-* or *P. ciferrii*-infected bMECs. Green fluorescence is expression of NLRP3 and ASC proteins. **C**–**G** Treatment with MT (10 µM) for 12 h inhibited expressions of NLRP3, Pro Caspase1, Caspase1 p20, and ASC proteins in *P. bovis-* or *P. ciferrii*-infected bMECs. “-” and “ + ” after MT indicated that MT was not or were added, respectively; “-” and “ + ” after *P. bovis* indicated that *P. bovis* was not or were added, respectively; “-” and “ + ” after *P. ciferrii* indicated that *P. ciferrii* was not or were added, respectively. Data represent means ± SD of 3 independent experiments. ^*^P < 0.05 or ^**^P < 0.01, difference compared to the control; ^#^*P* < 0.05 or ^##^*P* < 0.01, difference compared to the *P. ciferrii* infection group; ^&^*P* < 0.05 or ^&&^*P* < 0.01, difference compared to the *P. bovis* infection group.

The green fluorescence intensity of NLRP3 and ASC was higher after infection with *P. bovis* or *P. ciferrii* compared to the control (Figures 6A and B). In contrast, treatment with mito-TEMPO decreased the green fluorescence intensity of NLRP3 and ASC compared to infection with either *P. bovis* or *P. ciferrii* (Figures 6A and B). Furthermore, expression levels of NLRP3, Pro Caspase1, Caspase1 p20, and ASC proteins were upregulated in *P. bovis*-infected cells (*P* < 0.05) compared to the control (Figures 6C, D, E, F and G). Infection of bMECs with *P. ciferrii* also increased expression of these proteins, but there was no significant change compared to the control (lowest *P* = 0.14) (Figures 6C, D, E, F and G). Expression of these proteins were all upregulated after *P. bovis* or *P. ciferrii* infections, with more pronounced increases for *P. bovis*. However, in bMECs pretreated with mito-TEMPO, expression of NLRP3, Pro Caspase1, Caspase1 p20, and ASC proteins were inhibited in bMECs infected with *P. ciferrii* and *P. bovis* (except ASC, *P* < 0.05) (Figures 6C, D, E, F and G). Protein expression in NLRP3 inflammasome pathway was not significantly different among isolates within *P. bovis* or *P. ciferrii* species (Additional file 2).

### *Prototheca* spp. infection enhanced protein expression of IL-1β in bMECs

**Figure 7:**
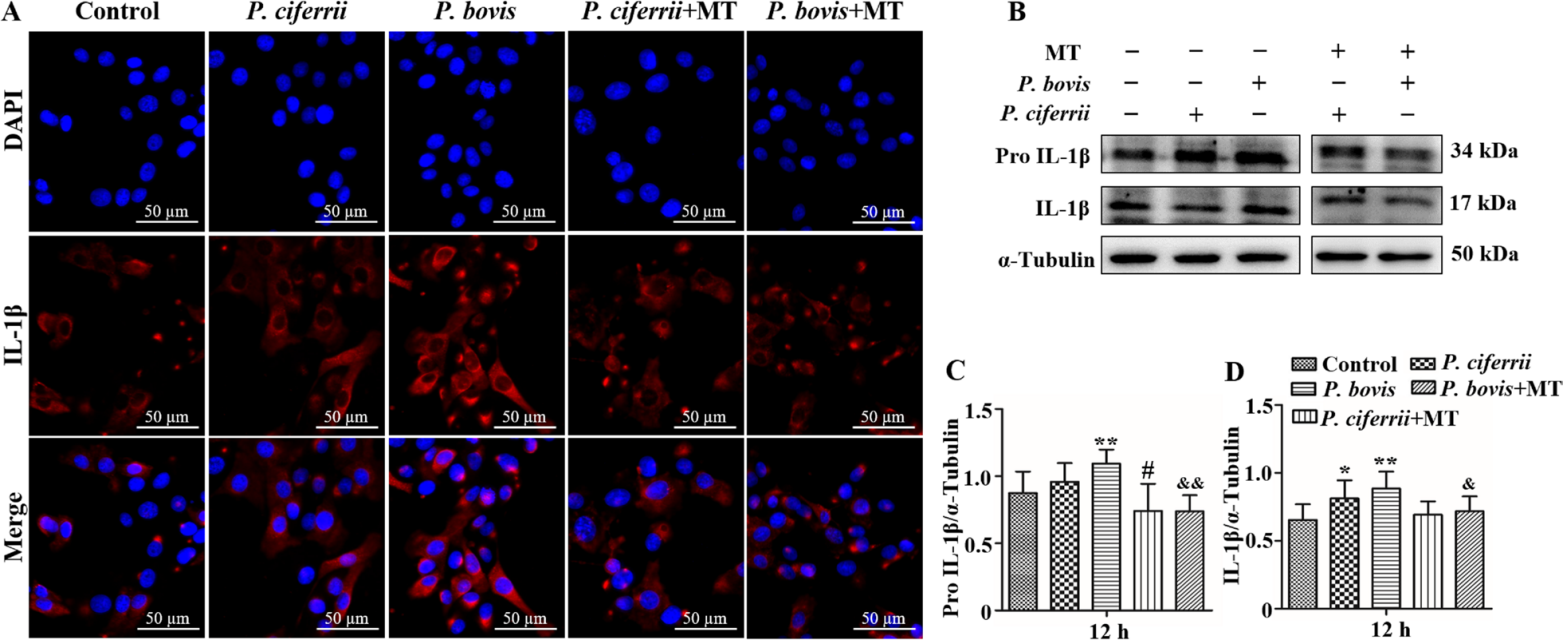
**Expression IL-1β protein in bMECs.** **A** Treatment with MT (10 µM) for 12 h inhibited IL-1β expression in *P. bovis-* or *P. ciferrii*-infected bMECs. Red fluorescence is expression of IL-1β protein. **B–D** Treatment with MT (10 µM) for 12 h inhibited expression of Pro IL-1β and IL-1β proteins in *P. bovis-* or *P. ciferrii*-infected bMECs. “-” and “ + ” after MT indicated that MT was not or were added, respectively; “-” and “ + ” after *P. bovis* indicated that *P. bovis* was not or were added, respectively; “-” and “ + ” after *P. ciferrii* indicated that *P. ciferrii* was not or were added, respectively. Data represent means ± SD of 3 independent experiments. ^*^P < 0.05 or ^**^P < 0.01, difference compared to the control; ^#^*P* < 0.05 or ^##^*P* < 0.01, difference compared to the *P. ciferrii* infection group; ^&^*P* < 0.05 or ^&&^*P* < 0.01, difference compared to the *P. bovis* infection group.

Infection with *P. bovis* or *P. ciferrii* increased the red fluorescence intensity of IL-1β in bMECs compared to the control (Figure 7A). However, treatment with mito-TEMPO decreased the red fluorescence intensity of IL-1β in bMECs infected with *P. bovis* or *P. ciferrii* (Figure 7A). Furthermore, expression of Pro IL-1β protein was upregulated in bMECs infected with *P. bovis* (*P* < 0.05) (Figures 7B and C). In addition, IL-1β protein was also upregulated in bMECs infected with *P. bovis* or *P. ciferrii* (*P* < 0.05) (Figures 7B and D). Expression of Pro IL-1β and IL-1β proteins were higher after *P. bovis* compared to *P. ciferrii* infection. Treatment with mito-TEMPO inhibited expression of Pro IL-1β after *P. bovis* infection (*P* < 0.05) and IL-1β protein in *P. bovis* and *P. ciferrii* infected bMECs were downregulated (*P* < 0.05) (Figures 7B, C and D). Protein expression of IL-1β was not significantly different among isolates within *P. bovis* or *P. ciferrii* species (Additional file 2).

## Discussion

In this study, *Prototheca* spp. infection in bMECs induced an inflammatory response through the NF-κB and NLRP3 inflammasome pathways. Infection of bMECs with *Prototheca* spp., especially *P. bovis*, damaged mitochondria and promoted mtROS accumulation, which activated an inflammatory response through the NF-κB and NLRP3 inflammasome pathways and enhanced IL-1β production. However, scavenging mtROS decreased expressions of proteins in NF-κB/NLRP3 inflammasome pathways and IL-1β production in bMECs infected with *P. bovis* or *P. ciferrii*. Accumulation of mtROS may be important in inflammatory responses to *P. bovis* or *P. ciferrii* infections. Furthermore, mtROS activated NF-κB/NLRP3 inflammasome pathways were involved in inflammation in bMECs infected with *P. bovis* and *P. ciferrii*.

Pathogenic infections can cause mitochondrial damage, including swelling and vacuolation, increase ROS, decrease membrane potential, and increase oxidative stress, both in vitro and in vivo [31–33]. Mitochondrial damage is closely related to development of inflammatory diseases [34]. In the current study, *Prototheca* spp. infections in bMECs, especially *P. bovis*, caused dissolution and large area vacuolation of mitochondrial cristae, and decreased mitochondrial activity. Mitochondria are the main site for mtROS production [35] and mitochondrial damage may contribute to mtROS production. Based on Mito-SOX, mtROS in bMECs increased after infection with *P. bovis*, with a lesser increase in *P. ciferrii*-infected bMECs. NADPH is closely linked to ROS production and there is increasing evidence that increases in both ROS production and expression of NADPH oxidase were upregulated both in vitro and in vivo [36, 37]. In the present study, *P. bovis* or *P. ciferrii* infections in bMECs increased NADPH, which also provided evidence for production of mtROS. In this study, although strains for each species (*P. bovis* and *P. ciferrii*) were isolated from different samples, the pathogenicity of strains in each species to bMECs was similar, as both *P. bovis* and *P. ciferrii* induced mitochondrial damage and mtROS accumulation, with the former causing more profound damage.

Increased mitochondrial ROS promoted inflammatory responses in peritoneal mesothelial cells, macrophages and T cells [15, 38]. Infection of bMECs with *P. bovis* or *P. ciferrii* stimulated inflammatory responses, characterized by release of inflammatory cytokines which activate immune effector cells to eliminate invading pathogens. There were significant increases in production of TNF-α, IL-18, and IL-1β at 12 h after bMECs were infected with *P. bovis* or *P. ciferrii*, indicating a marked inflammatory response. However, responses to *P. bovis* were more severe than *P. ciferrii*, consistent with its greater pathogenicity. However, scavenging mtROS with mito-TEMPO significantly decreased cytokine production. Therefore, we inferred that infection with *P. bovis* or *P. ciferrii* induced inflammatory responses in bMECs that were mitigated by suppression of mtROS.

Inflammatory responses have many regulatory mechanisms, including the NF-κB and NLRP3 inflammasome pathways [39, 40]. In the NF-κB pathway, both IκBα and NF-κB p65 are inactive in the cytoplasm [41, 42]. However, when an upstream signal activates inhibitor of nuclear factor kappa-B kinase (IKK), it will be ubiquitinated, phosphorylated and degrade IκBα, so that NF-κB p65 will be activated and translocated from the cytoplasm to the nucleus to bind to the corresponding inflammation-related genes, promote transcription of inflammatory cytokines, and induce inflammation [42, 43]. In the current study, infection of bMECs with *P. bovis* or *P. ciferrii* activated the NF-κB pathway, upregulating expression of IκBα and NF-κB p65 proteins. Furthermore, expression levels of phosphorylated IκBα and NF-κB p65 proteins were upregulated after *P. bovis* or *P. ciferrii* infection, with greater upregulation of protein expression in the NF-κB pathway induced by *P. bovis*, indicating higher pathogenicity. Activation of the NF-κB pathway promoted inflammation, including a massive increase in cytokine production. In addition, mtROS activation of the IKK complex and subsequent signaling through the NF-κB pathway led to secretion of proinflammatory cytokines by inducing the intermolecular disulfide linkage of nuclear factor IκBα essential modulator [44], whereas quenching mtROS in vivo decreased the NF-κB-guided anti-inflammatory phenotype [45]. In the present study, in bMECs infected with *P. bovis* or *P. ciferrii*, mito-TEMPO decreased expression of various proteins in the NF-κB pathway, including IκBα, NF-κB p65, p-IκBα and p-NF-κB p65. Thus, infection of bMECs with *Prototheca* spp., especially *P. bovis*, caused overexpression of proteins in the NF-κB pathway and enhanced inflammatory responses through generation of mtROS.

Activation of inflammasomes is critical in inflammatory responses, with key roles in regulating inflammation caused by pathogenic bacteria [46, 47]. The NLRP3 inflammasome is well characterized [48]. Once activated, ASC self assembles and activates Pro Caspase1; the activated Caspase1 induces maturation of IL-1β and IL-18 for subsequent release [48, 49]. Activation of the NLRP3 inflammasome is regulated by many factors, including bacterial infections and mtROS [50, 51]. In the present study, *P. bovis* or *P. ciferrii* infection in bMECs promoted activation of NLRP3 inflammasomes to varying degrees, modulating upregulation of expression of NLRP3, ASC, and Caspase1 proteins, and promoting cleavage of Caspase1. We inferred that infection with either *P. bovis* or *P. ciferrii* contributed to the assembly of ASC, Pro Caspase1, and NLRP3 during inflammasome formation. Although *P. bovis* or *P. ciferrii* infections in bMECs promoted NLRP3 inflammasome activation, *P. bovis* induced larger increases in proteins of the NLRP3 inflammasome, indicating greater pathogenicity. Furthermore, expression levels of proteins of the downstream genes Pro IL-1β and IL-1β were upregulated in cells infected with *P. bovis* or *P. ciferrii*, although *P. bovis* caused more pronounced increases. Therefore, *P. bovis* induced a greater inflammatory response than *P. ciferrii* vai the NLRP3 inflammasom pathway.

**Figure 8:**
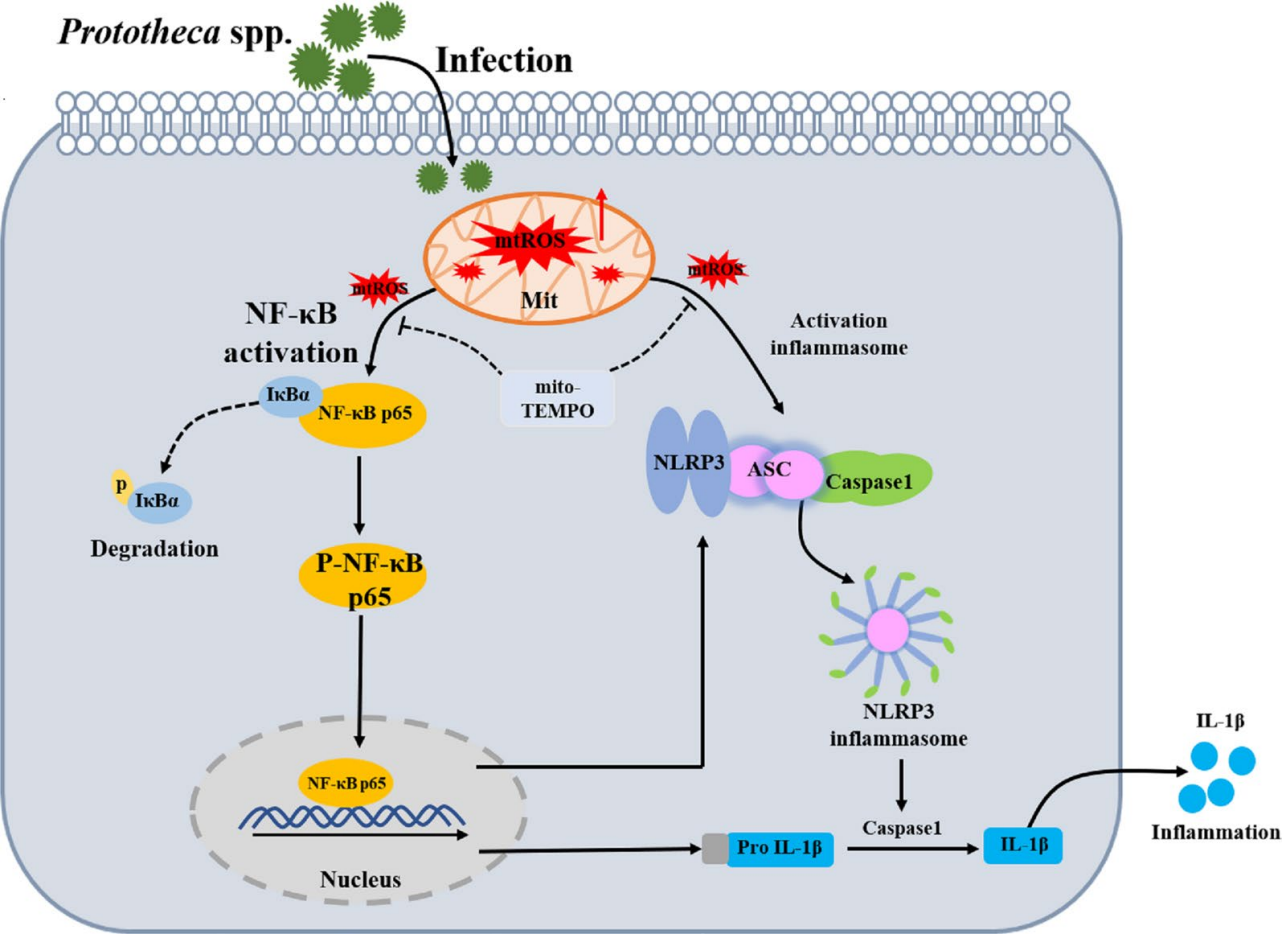
**The pattern of Prototheca spp. infection induced inflammatory responses through NF-κB and NLRP3 inflammasome pathways in bMECs.** Infection of bMECs with *P. bovis* or *P. ciferrii*, increased mtROS, which can promote activation of NF-κB and NLRP3 inflammasome pathways and further enhance production of IL-β, resulting in an inflammatory response in bMECs. However, in bMECs treated with mito-TEMPO, which scavenged mtROS, protein expression in NF-κB and NLRP3 inflammasome pathways was inhibited and there was suppression of the inflammatory response in *P. bovis-* or *P. ciferrii*-infected bMECs.

Generation of mtROS is one of the first identified triggers of NLRP3 inflammasome activation, although mtROS-independent activation of the NLRP3 inflammasome has been reported [52, 53]. In the present study, *P. bovis* or *P. ciferrii* infections induced mtROS in bMECs. However, treatment with mito-TEMPO downregulated expression levels of NLRP3, ASC and Caspase1 proteins in bMECs infected with *P. bovis* or *P. ciferrii*, whereas expression of Pro IL-1β and IL-1β proteins was also downregulated. Therefore, we inferred that mtROS has an important role in activation of NLRP3 inflammasomes and enhances production of IL-β, resulting in an inflammatory response in bMECs. Mito-TEMPO suppressed expression of proteins in NF-κB and NLRP3 inflammasomes pathways and reduced inflammatory responses in *P. bovis-* and *P. ciferrii*-infected bMECs (Figure 8). In this study, 3 strains in each species were randomly selected to infect bMECs. Consequently, we maximized the probability of choosing strains that represented other strains within each species in terms of pathogenicity. Regardless, mechanisms of *Prototheca* spp.-infected cells inducing mtROS generation need further study.

Infections of bMECs with either *P. bovis* or *P. ciferrii* damaged mitochondria and induced inflammatory responses, with *P. bovis* causing a more severe inflammatory response*.* Accumulation of mtROS had an important role in activation of NF-κB and NLRP3 inflammasomes and suppression of mtROS reduced inflammatory responses in bMECs infected with either *P. bovis* or *P. ciferrii*.

## Supplementary Information

**Additional file 1. The coefficient of variation values (ELISA and Real time PCR) of P. ciferrii and P. bovis infections of bMECs.** The coefficient of variation (cv) is the standard deviation divided by the mean. We calculated the cv values among biological replicates within *P. bovis* and *P. ciferrii*, respectively. These cv values reflect the variation among different biological replicates and the cv values were in the range of 0.001–0.098, therefore we consider the variation among biological replicates was acceptable.

**Additional file 2. The coefficient of variation values (Western blot) of P. ciferrii and P. bovis infections of bMECs.** The coefficient of variation (cv) is the standard deviation divided by the mean. We calculated the cv values among biological replicates within *P. bovis* and *P. ciferrii*, respectively. These cv values reflect the variation among different biological replicates and the cv values were in the range of 0.006–0.298, therefore we consider the variation among biological replicates was acceptable.

## Data Availability

All data generated or analyzed during this study are included in this published article.

## Abbreviations

bMECs: bovine mammary epithelial cells
SDA: sabouraud dextrose agar
SDB: sabouraud dextrose broth
NADPH: nicotinamide adenine dinucleotide phosphate
mtROS: mitochondrial reactive oxygen species
mito-TEMPO: mitochondrial specific antioxidant
CCK-8: Cell Counting Kit-8
BCA: Bicinchoninic acid
RIPA: radioimmunoprecipitation assay
BSA: bovine serum albumin
DAPI: 4’, 6-Diamidine-2’-phenylindole dihydrochloride
PVDF: polyvinylidene difluoride
ECL: enhanced chemiluminescence
FBS: fetal bovine serum
DMEM: Dulbecco’s Modified Eagle’s medium
PBS: phosphate buffered solution
MOI: multiplicity of infection
TEM: transmission electron microscopy
