# Supplementary material for: Prototheca spp. induce an inflammatory response via mtROS-mediated activation of NF-κB and NLRP3 inflammasome pathways in bovine mammary epithelial cell cultures
Source: Vet Res. 2021 Dec 11;52:144. doi: 10.1186/s13567-021-01014-9 (PMC8666081; doi:10.1186/s13567-021-01014-9)
Supplement: Supplementary file 1 — Additional file 1. The coefficient of variation values (ELISA and Real time PCR) of P. ciferrii and P. bovis infections of bMECs. The coefficient of variation (cv) is the standard deviation divided by the mean. We calculated the cv values among biological replicates within P. bovis and P. ciferrii, respectively. These cv values reflect the variation among different biological replicates and the cv values were in the range of 0.001–0.098, therefore we consider the variation among biological replicates was acceptable. [file 13567_2021_1014_MOESM1_ESM.docx]

**Additional file 1A The coefficient of variation values (ELISA) of *P. ciferrii* and *P. bovis* infections of bMECs.**

|  | *P. ciferrii* | *P. bovis* | *P. ciferrii*+MT | *P. bovis* +MT |
| --- | --- | --- | --- | --- |
| IL-1β | 0.011 | 0.007 | 0.008 | 0.017 |
| IL-18 | 0.022 | 0.024 | 0.014 | 0.015 |
| TNF-α | 0.037 | 0.040 | 0.034 | 0.039 |

**Additional file 1B The coefficient of variation values (Real time PCR) of *P. ciferrii* and *P. bovis* infections of bMECs.**

|  | *P. ciferrii* | *P. bovis* | *P. ciferrii*+MT | *P. bovis* +MT |
| --- | --- | --- | --- | --- |
| IL-1β | 0.013 | 0.019 | 0.001 | 0.073 |
| IL-18 | 0.098 | 0.064 | 0.033 | 0.016 |
| TNF-α | 0.020 | 0.017 | 0.028 | 0.053 |
