# Supplementary material for: Prototheca spp. induce an inflammatory response via mtROS-mediated activation of NF-κB and NLRP3 inflammasome pathways in bovine mammary epithelial cell cultures
Source: Vet Res. 2021 Dec 11;52:144. doi: 10.1186/s13567-021-01014-9 (PMC8666081; doi:10.1186/s13567-021-01014-9)
Supplement: Supplementary file 2 — Additional file 2. The coefficient of variation values (Western blot) of P. ciferrii and P. bovis infections of bMECs. The coefficient of variation (cv) is the standard deviation divided by the mean. We calculated the cv values among biological replicates within P. bovis and P. ciferrii, respectively. These cv values reflect the variation among different biological replicates and the cv values were in the range of 0.006–0.298, therefore we consider the variation among biological replicates was acceptable. [file 13567_2021_1014_MOESM2_ESM.docx]

**Additional file 2 The coefficient of variation values (Western blot) of *P. ciferrii* and *P. bovis* infections of bMECs.**

|  | *P. ciferrii* | *P. bovis* | *P. ciferrii*+MT | *P. bovis* +MT |
| --- | --- | --- | --- | --- |
| P65 | 0.086 | 0.062 | 0.106 | 0.033 |
| P-p65 | 0.033 | 0.046 | 0.252 | 0.192 |
| IκB | 0.119 | 0.082 | 0.060 | 0.006 |
| P- IκB | 0.221 | 0.139 | 0.050 | 0.094 |
| NLRP3 | 0.058 | 0.019 | 0.232 | 0.298 |
| Caspase1 | 0.121 | 0.081 | 0.074 | 0.028 |
| Caspase1 p20 | 0.092 | 0.120 | 0.103 | 0.131 |
| ASC | 0.207 | 0.243 | 0.273 | 0.146 |
| Pro IL-1β | 0.055 | 0.053 | 0.239 | 0.140 |
| IL-1β | 0.106 | 0.094 | 0.081 | 0.015 |
